# Fragmentation of Deprotonated Diacylhydrazine Derivatives in Electrospray Ionization Tandem Mass Spectrometry: Generation of Acid Anions via Intramolecular Rearrangement

**DOI:** 10.1371/journal.pone.0063097

**Published:** 2013-05-21

**Authors:** Kezhi Jiang, Hu Zhang, Jianmei Wang, Fei Li, Mingrong Qian

**Affiliations:** 1 Key Laboratory of Organosilicon Chemistry and Material Technology, Hangzhou Normal University, Hangzhou, Zhejiang, China; 2 MOA Key Lab for Pesticide Residue Detection, Institute of Quality and Standard for Agro-products, Zhejiang Academy of Agricultural Sciences, Hangzhou, Zhejiang, China; University of Calgary, Canada

## Abstract

The gas-phase fragmentation pathways of deprotonated diacylhydrazine derivatives (R_1_(C = O)-N(*t-*Bu)NH(C = O)R_2_, Compounds **1**–**6**) were investigated by the combination of electrospray ionization tandem mass spectrometry (ESI-MS/MS) and theoretical calculations. Upon collisional activation, the deprotonated molecular ions [M – H]^−^ dissociate in two reaction channels, both of which involve intramolecular rearrangement. The main product ion is confirmed to be an anionic acid species, [R_1_-CO_2_]^−^, generated through intramolecular rearrangement of [M – H]^−^ initiated by the nucleophilic attack of the amide O6 on the carbonyl C2 (Path-1). The minor fragment channel (Path-2) involves methylpropene elimination of the precursor ion, followed by a similar nucleophilic displacement reaction to produce another acid anion [R_2_-CO_2_]^−^. Density functional theory calculations at the B3LYP/6-31+G(d,p) level indicate that Path-1 is more favorable than Path-2 for dissociation of the deprotonated halofenozide.

## Introduction

**Figure 1:**
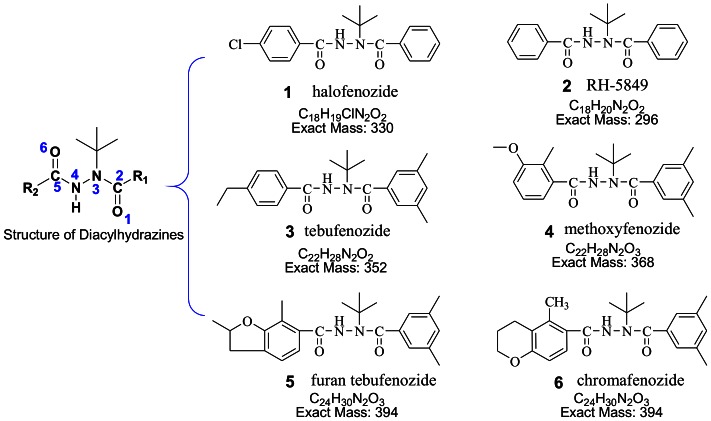
Structures for the deveratives of diacylhydrazine.

Derivatives of diacylhydrazine (Figure 1) were widely-used as insect growth regulators against *Lepidoptera* pests in agriculture, which induce premature molting and cause insect death by mimicking their hormones [1]–[5]. To ensure food safety, methods have been developed to determine and quantify these diacylhydrazine residues in fruits and vegetables, of which liquid chromatography-electrospray tandem mass spectrometry (HPLC-ESI-MS/MS) is the most powerful and widely-used analytical tool [6]–[13]. These compounds, other than halofenozide, were mainly investigated by the positive ion ESI tandem mass spectrometry, and the results showed that the major dissociation reactions of protonated diacylhydrazines observed in MS/MS were the cleavage of the amide bond and the loss of methylpropene from the *tert*-butyl group via proton transfer [6]–[10], [12]. A few recent studies of diacylhydrazines were also reported to be detected in the negative ion mode [11], [13]. However, a mechanistic explanation of the fragmentation pathways for the formation of these product ions has not yet been provided.

Deprotonated molecules of many important species were generated in the negative ion mass spectrometry, and they underwent charge induced and charge-remote fragmentations in the tandem mass spectrometry, which were much different from the fragmentation behaviors of the corresponding protonated species [14]–[21]. Many negative ions were favorable to undergo the intramolecular nucleophilic displacement, which led to unusual fragment ions [22]–[28]. The characteristic fragmentations provided useful information for structural elucidation [28]–[32] and differentiation of isomers [15], [21]. Obviously, the title compounds can be easily ionized in the negative mode, due to the presence of the amide hydrogen function [18]. However, the major fragment ions observed in the negative ion MS/MS spectra cannot be interpreted by the direct dissociation [11], [13]. The purpose of the present work is to perform a detailed mechanistic investigation of the fragmentation pathways of the deprotonated diacylhydrazines in the negative ion mode.

Density functional theory (DFT) calculations have proved to be excellent for rationalization of mass spectrometric fragmentation [33]–[34], and a joint of theoretical calculation and high-resolution MS measurement have become essential methods for mechanistic investigation on the fragmentation of the gas-phase ions [35]. The B3LYP functional with the basis set 6−31+G(2d,p) for geometry optimizations has been widely used, due to its accuracy and the computationally moderate time consumption [36]. In this paper, accurate MS measurement and DFT calculations were performed to probe the mechanistic fragmentation of the deprotonated diacylhydrazine pesticides. The results indicate a novel skeletal rearrangement of the deprotonated molecules prior to fragmentation.

## Experiments

### Chemicals

RH-5849 (*N′*-benzoyl-*N-tert*-butylbenzohydrazide, purity 95.0%) was provided by the Institute for Control of Agrochemicals (China). Five analytical standards in Figure 1 (purity 95.0% or better) were purchased from Dr. Ehrenstorfer (Augsburg, Germany). Methanol (HPLC grade) was obtained from Merck (Darmstadt, Germany). Purified water was prepared by using a Milli-Q water purification system (Millipore Purification Systems).

### Mass Spectrometry

The MS/MS experiments were performed on an LCQ Advantage quadruple ion trap mass spectrometer (Thermo-Fisher Scientific Inc., USA). The diluted diacylhydrazine solutions were directly infused into the electrospray ionization source of the mass spectrometer with a syringe pump at a flow rate of 5 µL/min. The sheath gas (N_2_, 99.99%) was set at 25 arbitrary units (a.u.). The spray voltage was set at −4.5 kV, and the heated ion transfer tube (250°C) was set at +55 V for ion introduction. The ion trap pressure (about 3×10^−3^ Pa) was maintained with a Turbo pump and pure helium (99.999%), was used for the trapping and collision activation of the selected ions. Full scan experiments were performed in the ion trap in the range of *m/z* of 90 to 450. Isolation width in the MS/MS experiment was set at 1.2 *m/z* and the activation time at 30 ms. The RF activation amplitude was set at 28% of the 5 V applied to the end cap electrodes to effect dissociation. Data acquisition and analysis were performed using the Xcalibur software package (Version 2.0 SR1).

Accurate MS of the product ions were measured by a microTOF QII (Q-TOF) mass spectrometer (Bruker Company, USA), equipped with an ESI ion source. The diluted diacylhydrazine solutions were also directly infused into the ESI source with a syringe pump at a flow rate of 180 µL/hr. The parameters of the Q-ToF mass spectrometer were set as follows: N_2_ drying gas flow in the ion source, 4 L/hr; N_2_ nebulizer gas pressure, 0.4×10^5^ Pa; ion source temperature, 180°C; ion source voltage, 3.5 kV; and CID collision energy for the selected ions was 15 eV with Ar as collision gas. The instrument was operated at a resolution higher than 15 000 full width at half maximum using the micrOTOF-Q Control ver. 2.3 control program. Data were analyzed using the Data Analysis ver. 4 software package delivered by Bruker Daltonics. The data were acquired as a continuous cycle of MS and MS/MS acquisitions of the five most intense peaks.

### Theoretical Calculation

The theoretical calculations were performed using the Gaussian 03 program [36]. The equilibrium geometries of the target species were optimized using the DFT method at the B3LYP/6−31+G(2d,p) level. The optimized structures were identified as the true energy minima by the absence of imaginary frequencies. Transition states, on the other hand, were identified by the presence of one single imaginary vibration frequency and the normal vibrational mode. The transition states were further confirmed using the intrinsic reaction coordinates (IRC) calculations. The optimized structures were visualized with the GaussView (Version 3.09) software. Hard data on geometries of all the structures involved are available in Tables S1–S18 in Text S1 of the Supporting Information.

## Results and Discussion

### ESI-MS^2^ Analysis

**Figure 2:**
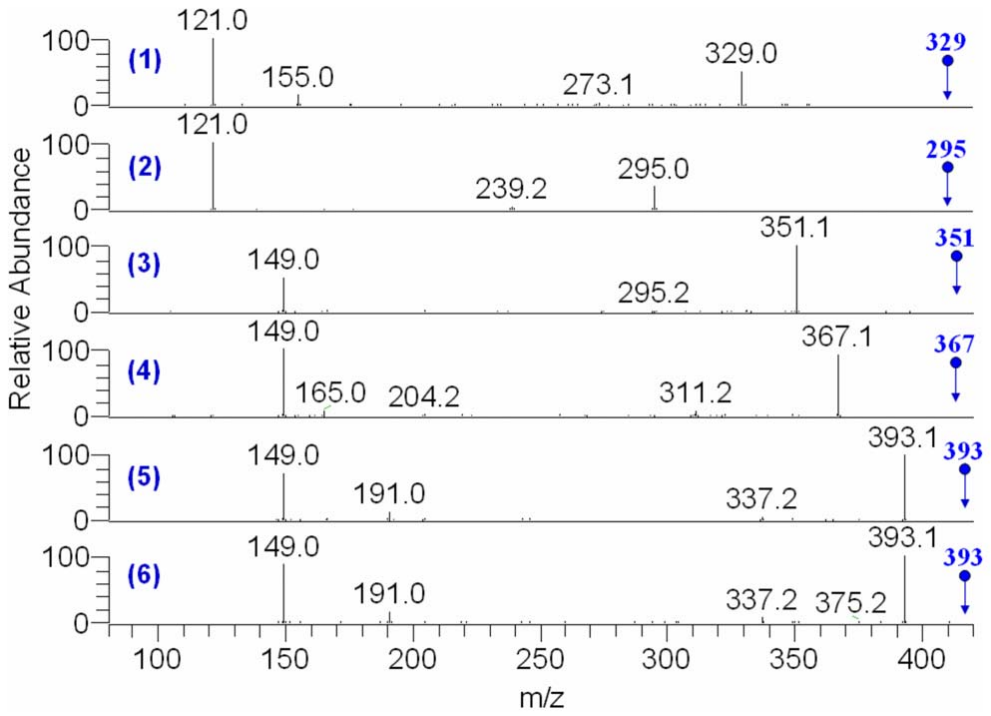
MS/MS of the deprotonated compounds 1–6 measured by ESI-IT MS at the normalized collision energy of 25%.

**Figure 3:**
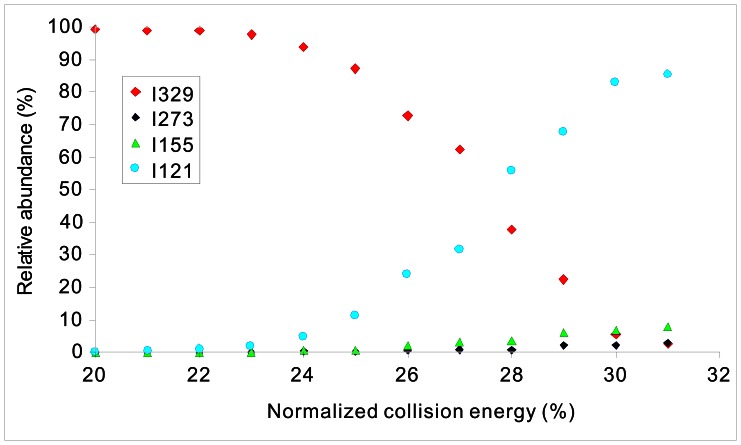
Energy-resolved plot (Relative abundance I_i_/∑I_i_ *VS* Normalized collision energy) of the deprotonated Halofenozide (*m/z* 329), by an LCQ IT-MS spectrometer.

Figure 2 displays the CID mass spectra of the deprotonated diacylhydrazines under negative ion ESI conditions as described in the experimental section, and the corresponding results are summarized in Table 1. Generally, the deprotonated ions [M – H]^−^ of Compounds **1**–**6** displayed similar fragmentation patterns upon collisional activation; specifically the species participated in dissociation reactions which mainly generated one major product ion and two minor fragment ions. Moreover, analysis of the collisional energy-resolved plot for the deprotonated halofenozide in Figure 3 indicates that dissociation of the precursor ion (*m/z* 329) mainly generated the major abundant fragment ion at *m/z* 121, and two minor fragment ions at *m/z* 273 and *m/z* 155 with various relative collision energies ranging from 20% to 31%.

**Table 1:** The CID-MS results of the deprotonated ions [M – H]^−^ for compounds 1–6.

	[M – H]^−^	Product ions, *m/z* (RA%)		
Compound	*m/z* (RA%)	[M – H–56]^−^	Main product ion [R_1_-CO_2_]^−^	Minor product ion [R_2_-CO_2_]^−^
**1**	329 (50.3%), ^35^Cl	273 (5.5%)	121 (100%)	155 (16.6%)
	331 (56.2%), ^37^Cl	275 (3.7%)	121 (100%)	157 (10.2%)
**2**	295 (34.9%)	239 (3.8%)	121 (100%)	–
**3**	351 (100%)	295 (1.8%)	149 (51.3%)	–
**4**	367 (92.9%)	311 (5.7%)	149 (100%)	165 (8.0%)
**5**	393 (100%)	337 (3.1%)	149 (69.5%)	191 (11.7%)
**6**	393 (100%)	337 (7.4%)	149 (87.2%)	191 (15.7%)

As shown in Figure 2, the fragment ion with higher *m/z* is 56 Da lower in mass than the corresponding precursor ion. All the diacylhydrazines studied (depicted in Figure 1) share similar structures, in which one of the amide nitrogen atoms (N3) is bonded to a *tert-*butyl group and the other (N4) to a hydrogen atom. Methylpropene (56 Da) elimination was reported to be the main fragmentation pathway for the protonated species in the ESI tandem MS experiments [6]–[12]. Thereby, we also attribute the neutral fragment of 56 Da here to be methylpropene, coming from the *tert-*butyl group. Due to the relatively low abundance of the fragment ions in the accurate mass spectra obtained by Q-TOF, the relative errors of the masses measured and the masses calculated are about 10 ppm for the fragment ions [M – H–56]^−^ (Table 2).

**Table 2:** Comparison of the results of accurate mass determinations by Q-TOF and the masses calculated for the proposed ion structure of main product ions of deprotonated diacylhydrazines.

Compound	Measured mass	Relative intensity	Ion formula	Calculated mass	Relative error (ppm)
**1**	329.1069	35.5%	C_18_H_18_ClN_2_O_2_^−^	329.1062	−1.9
	273.0412	1.7%	C_14_H_10_ClN_2_O_2_^−^	273.0396	−5.9
	154.9904	4.5%	C_7_H_4_ClO_2_^−^	154.9905	0.7
	121.0293	100%	C_7_H_5_O_2_^−^	121.0295	1.5
**2**	295.1454	28.0%	C_18_H_19_N_2_O_2_^−^	295.1452	−0.7
	239.0799	1.0%	C_14_H_11_N_2_O_2_^−^	239.0826	11.1
	121.0293	100%	C_7_H_5_O_2_^−^	121.0295	1.7
**3**	351.2076	100%	C_22_H_27_N_2_O_2_^−^	351.2078	0.6
	295.1440	9.7%	C_18_H_19_N_2_O_2_^−^	295.1452	4.1
	149.0601	60.8%	C_7_H_5_O_2_^−^	149.0608	4.5
**4**	367.2029	65.7%	C_20_H_27_N_2_O_3_^−^	367.2027	−0.6
	165.0547	2.2%	C_9_H_9_O_3_^−^	165.0530	−10.3
	149.0604	100%	C_7_H_5_O_2_^−^	149.0608	2.7
**5**	393.2189	100%	C_24_H_29_N_2_O_3_^−^	393.2184	−1.5
	149.0601	91.5%	C_9_H_9_O_2_^−^	149.0608	4.8
**6**	393.2187	100%	C_24_H_29_N_2_O_3_^−^	393.2184	−0.8
	149.0601	81.5%	C_9_H_9_O_2_^−^	149.0608	4.8

**Figure 4:**
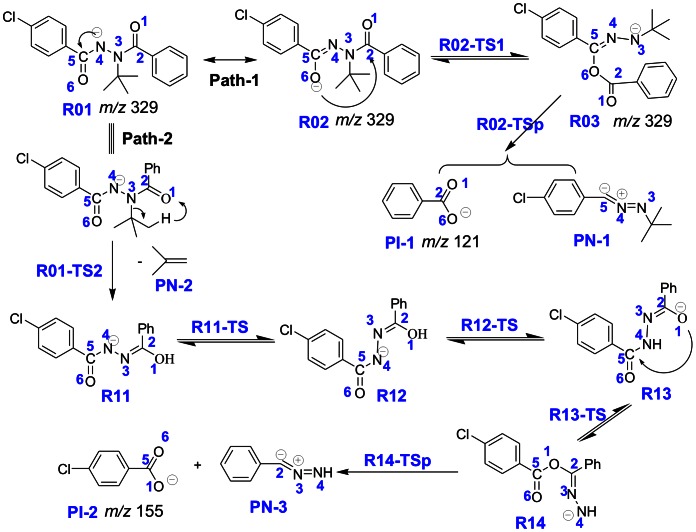
The proposed fragmentation pathways of the deprotonated halofenozide.

It is noteworthy that the mass of the major product ions is directly related to the R1 group structure. For compounds **1** and **2,** in which the benzoyl group (106 Da) is attached to the *tert-*butyl amide N3 (R_1_ is phenyl), the most abundant product ion is at *m/z* 121, and was postulated to be the benzoate anion [C_6_H_5_CO_2_]^−^. Whereas for compounds **3**–**6**, in which the 3,5-dimethylbenzoyl group (133 Da) bonds to N3 (R_1_ is 3,5-dimethylphenyl), the major product ion is at *m/z* 149, and was attributed to the 3,5-dimethylbenzoate anion [(CH_3_)_2_C_6_H_3_CO_2_]^−^. The elemental compositions of these three product ions have been confirmed by determining their accurate masses using high resolution mass spectrometer (Table 2, and Figures S1–S6 in the supporting information). The relative errors of the masses measured and the masses calculated from the assigned structures are less than 5 ppm for the precursor ions and the major fragment ions. That is, formation of the major fragment ion, [R_1_CO_2_]^−^, indicates migration of an O atom from the amide O6 to the amide C2 during the intramolecular rearrangement process (Figure 4).

Interestingly, the mass of the minor fragment ions with lower *m/z* is also 16 Da more than that of the acyl group (R_2_CO) bound to the amide N4 for compounds **1** and **4**–**6**, and these fragments were proposed to be another acid anion [R_2_CO_2_]^−^. The proposed structure of some of the minor fragment ions is further supported by the accurate mass analysis using ESI-Q-TOF (Table 2). Moreover, for compounds **2**–**3**, in which the R_1_ and R_2_ groups have the same mass, fragmentation of [M–H]^−^ produced one main and one minor product ion [M–H–56]^−^. Accordingly, formation of the minor fragment ion, [R_2_CO_2_]^−^ was proposed to undergo intramolecular rearrangement by transferring the amide O1 atom to the amide C5 (Figure 4).

### Fragmentation Pathways

**Figure 5:**
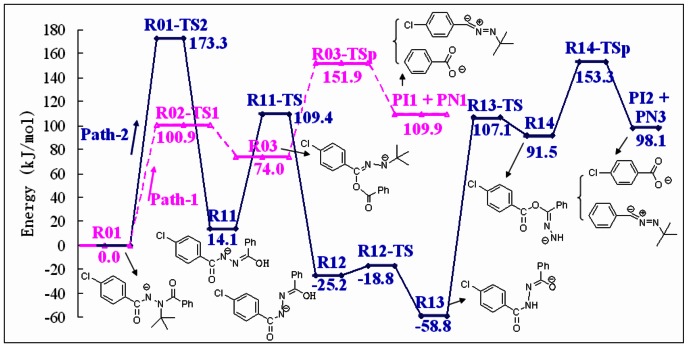
Reaction profile for the fragmentation of the deprotonated compound 1.

**Figure 6:**
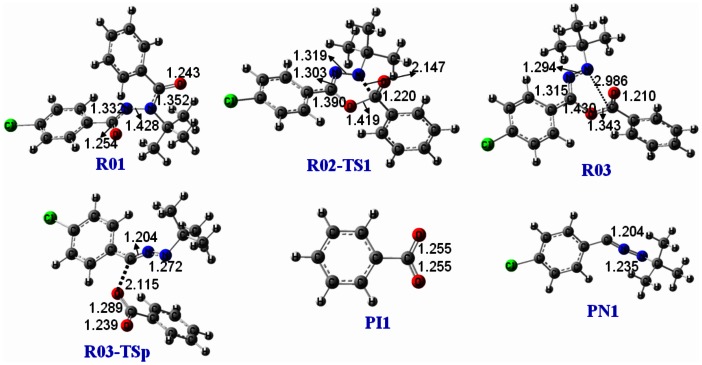
Optimized structures of the key species involved in the fragmentation pathway of Path-1 at B3LYP/6-31+G(2d,p).

**Figure 7:**
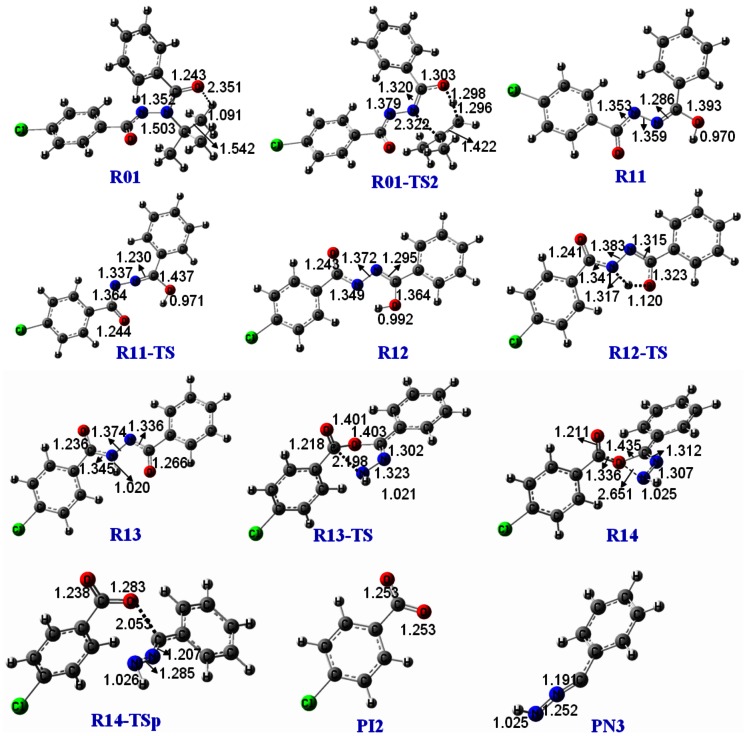
Optimized structures of the key species involved in the fragmentation pathway of Path-2 at B3LYP/6-31+G(2d,p).

Dissociation of the deprotonated diacylhydrazines is prone to undergo intramolecular rearrangement and generate the corresponding benzoic acid anion, according to the above MS/MS experimental results. Halofenozide (Compound **1**) was selected as the typical compound to explain the possible fragmentation pathways, since its main product ion at *m/z* 121 has been used as the quantification ion in previous LC-ESI(-)-MS/MS experiments [11], [13]. Under the negative ion ESI conditions, the deprotonated halofenozide [**1**– H]^−^ at *m/z* 329 was obtained by the loss of the active amide proton. As shown in Figure 4, the stability of [**1**– H]^−^ is a consequence of resonance, which disperses the negative charge to two centers, the amide N4 (**R01**) and the carbonyl O6 (**R02**). DFT calculations were performed at the B3LYP/6-31+G(2d,p) level of theory to probe the mechanism of the rearrangement reaction for the typical deprotonated halofenozide (Figure 4). The schematic potential energy diagrams for the reactions are shown in Figure 5, and full details of the structures of the species involved are provided in Figure 6 and Figure 7.

The amide O6 contains a partial negative charge in **R01** due to the resonance stability of the deprotonated anion, and **R01** is favorable to undergo intramolecular rearrangement initiated by the nucleophilic displacement in the gas phase. In the Path-1 reaction channel, nucleophilic attack of O6 at the carbonyl C2, leads to the breakage of the C2-N3 bond, which results in a benzyl ester intermediate **R03**. As shown in Figure 6, the transition state **R02-TS1** in the intramolecular nucleophilic displacement has a five-membered ring reaction center, with the forming O6-C2 bond (1.419 Å) and the breaking C2-N3 bond (2.147 Å). The Gibbs energy barrier for the conversion of **R01** into **R03** is only 100.9 kJ/mol, indicating a feasible process, and **R03** lies 74.0 kJ/mol above **R01** in Gibbs energy. The subsequent dissociation of **R03** leads to the main product ion of benzoic anion (**PI-1**, *m/z* 121) via breakage of the C5-O6 bond, as indicated by the C5-O6 bond length extension (1.430 Å in **R03** and 2.115 Å in **R03-TSp**). Dissociation of **R03** is the rate-determining step in the reaction channel of Path 1, and the calculated energy barrier via **R03-TSp** is 151.9 kJ/mol, relative to **R01**. The total Gibbs energy of **PI-1** and **PN-1** is 109.9 kJ/mol higher than **R01**.

For structure **R01**, one of the protons on the *tert-*butyl group can migrate to the carbonyl O1 in Path-2, which leads to methylpropene elimination via a six-membered ring transition state **R01-TS2** (Figure 7), and generates the minor fragment ion **R11** ([M–H–56]^−^) at *m/z* 273. The energy barrier for the process is 173.3 kJ/mol in Gibbs energy, and the total energy of **R11** and methylpropene lies 14.1 kJ/mol above **R01**. **R11** can then undergo a series of rearrangement reactions, and eventually gives the minor fragment ion at *m/z* 155 (**PI-2**).

The isomerization of **R11** at the C2 = N3 double bond to give **R12** is a feasible process, which surmounts an energy barrier of 95.3 kJ/mol, with exoergic by 39.3 kJ/mol. The subsequent migration of the enol proton from O1 to the deprotonated amide N4 in the **R12** structure leads to conversion to **R13**, a processes which is exoergic by 33.6 kJ/mol with a slight energy barrier of 6.4 kJ/mol. In fact, **R13** is at the global minimum of our calculated potential energy surface, with 72.9 kJ/mol lower than **R11** in Gibbs energy. **R13** then undergoes an intramolecular displacement reaction similar to Path-1, in which nucleophilic attack by the deprotonated amide O1 at C5 forms an anionic species of 4-chlorobenzoate, **R14**, via breakage of the C5-N4 bond. This nucleophilic displacement step has an energy barrier of 165.9 kJ/mol. The subsequent dissociation of **R14** results in formation of the 4-chlorobenzoate anion (**PI-2**, *m/z* 155), which surmounts an energy barrier of 61.8 kJ/mol via **R14-TSp**.

Analysis of the reaction profile in Path-2 indicates that methylpropene elimination of **R01** via **R01-TS2** is the key step in this reaction channel, which is 20.0 kJ/mol higher in energy than the rate-determining step (**R14-TSp**) of the subsequent fragmentation reaction pathway. Thereby, the generated product ion **R11** can easily undergo subsequent dissociation in the ESI tandem mass spectrometer. Furthermore, the energy barrier for Path-2 is 21.4 kJ/mol higher than that for Path-1. Path-1 has fewer activation steps in the reaction channel than Path-2, and hence Path 1 is the main channel in the fragmentation of **R01**. The calculated results are in a good qualitative agreement with the MS data.

### Conclusion

Dissociation of deprotonated diacylhydrazine derivatives, (R_1_(C = O)-N(*t-*Bu)- NH(C = O)R_2_, was studied by electrospray ionization tandem mass spectrometry (ESI-MS/MS) and theoretical calculations. The main product ion was confirmed to be the acid anion [R_1_-CO_2_]^−^, generated by gas-phase rearrangement of the deprotonated ion via nucleophilic attack of the amide O6 at the carbonyl C5 (Path-1). The minor fragment channel (Path-2) involves methylpropene elimination from the precursor ion, followed by a series of isomerization reactions and a similar nucleophilic displacement reaction to give another acid anion [R_2_-CO_2_]^−^. The results of DFT calculations at the B3LYP/6-31+G(2d,p) level indicated that Path-1 is more favorable than Path-2 for dissociation of the typical deprotonated halofenozide. Overall, the deprotonated diacylhydrazines were found to dissociate through a novel skeletal rearrangement pathway as determined by MS/MS analysis, and theoretical calculations were used to investigate the mechanism of the reaction.

## Supporting Information

Figure S1 **MS/MS of the deprotonated compound 1 measured by ESI-QTOF.**
(TIF)

Figure S2 **MS/MS of the deprotonated compound 2 measured by ESI-QTOF.**
(TIF)

Figure S3 **MS/MS of the deprotonated compound 3 measured by ESI-QTOF.**
(TIF)

Figure S4 **MS/MS of the deprotonated compound 4 measured by ESI-QTOF.**
(TIF)

Figure S5 **MS/MS of the deprotonated compound 5 measured by ESI-QTOF.**
(TIF)

Figure S6 **MS/MS of the deprotonated compound 6 measured by ESI-QTOF.**
(TIF)

Text S1 **Geometries for R01, R02-TS1, R03, R03-TSp, PI1, PN1, R01-TS2, R11, PN2, R11-TS, R12, R12-TS, R13, R13-TS, R14, R14-TSp, PI2, PN3.**
(DOC)
