# Supplementary figures and images for: Fragmentation of Deprotonated Diacylhydrazine Derivatives in Electrospray Ionization Tandem Mass Spectrometry: Generation of Acid Anions via Intramolecular Rearrangement
Source: PLoS One. 2013 May 21;8(5):e63097. doi: 10.1371/journal.pone.0063097 (PMC3660572; doi:10.1371/journal.pone.0063097)

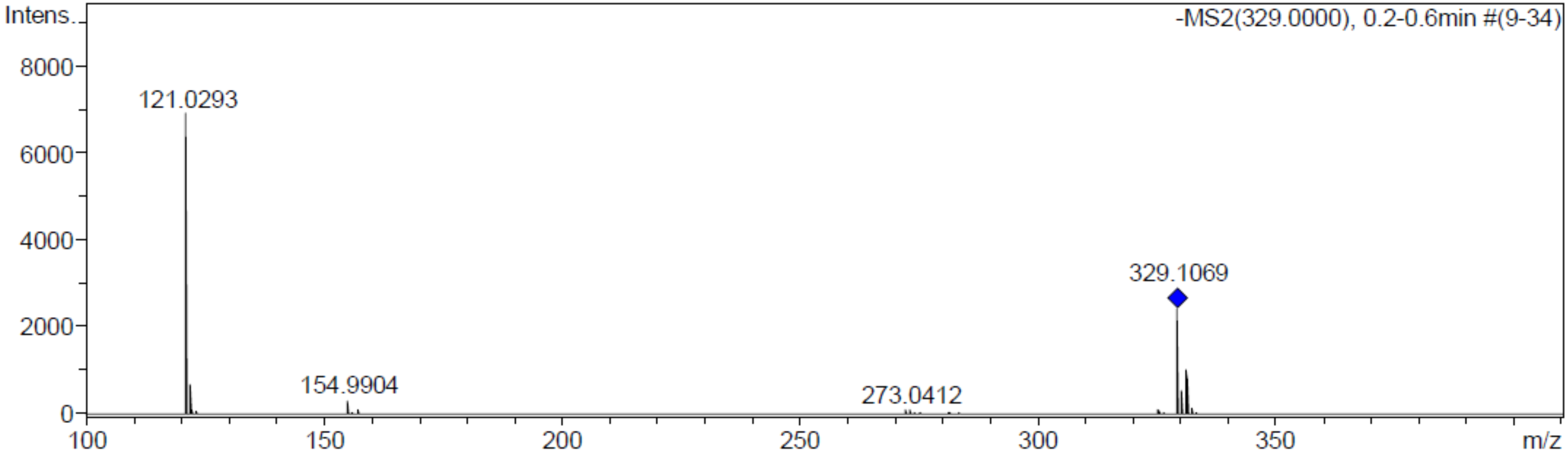

Supplement: Figure S1 — MS/MS of the deprotonated compound 1 measured by ESI-QTOF. (TIF) [file pone.0063097.s001.tif]

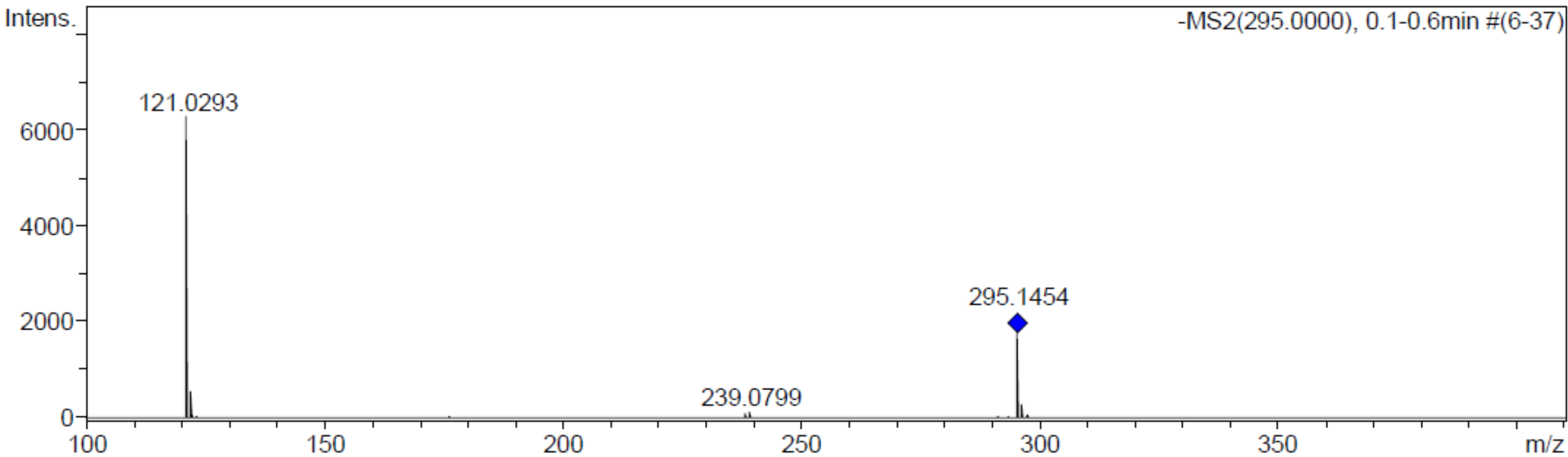

Supplement: Figure S2 — MS/MS of the deprotonated compound 2 measured by ESI-QTOF. (TIF) [file pone.0063097.s002.tif]

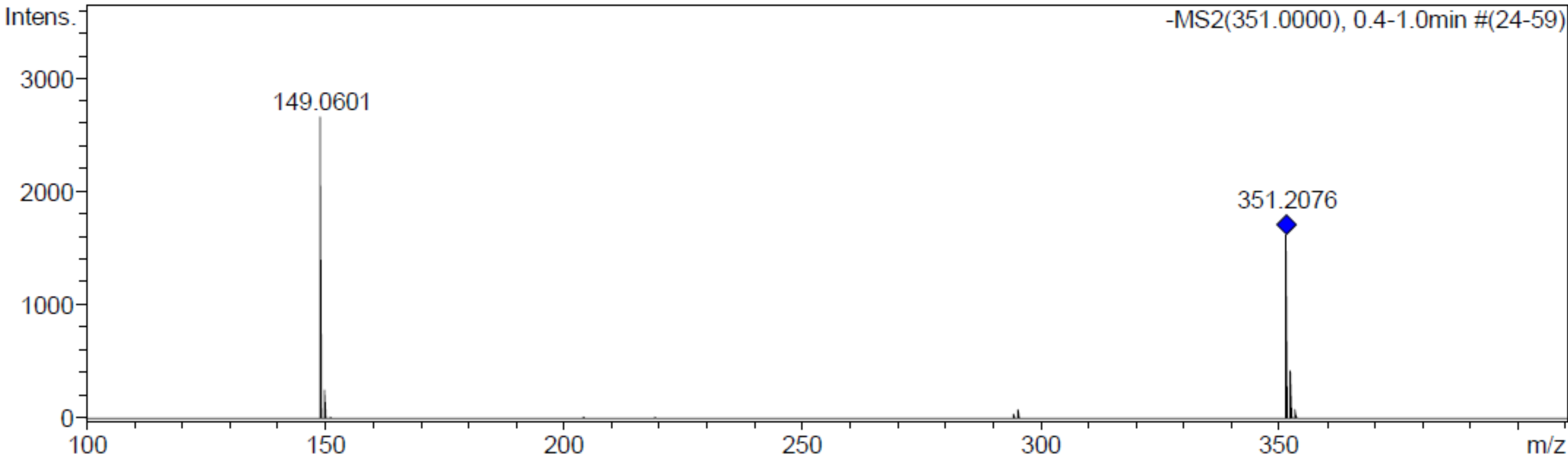

Supplement: Figure S3 — MS/MS of the deprotonated compound 3 measured by ESI-QTOF. (TIF) [file pone.0063097.s003.tif]

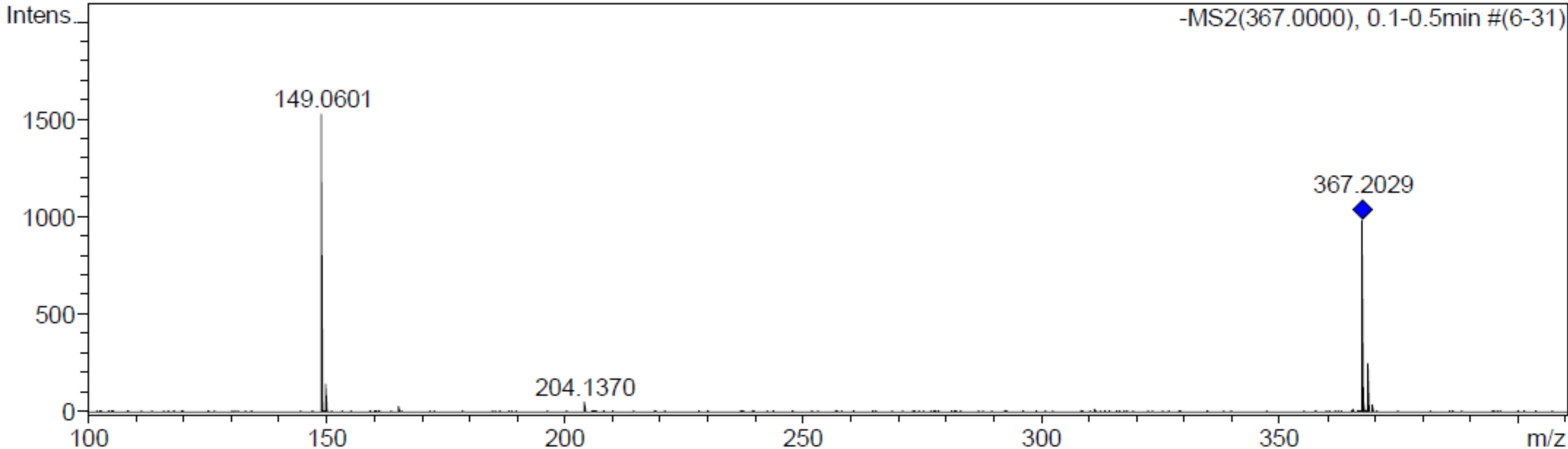

Supplement: Figure S4 — MS/MS of the deprotonated compound 4 measured by ESI-QTOF. (TIF) [file pone.0063097.s004.tif]

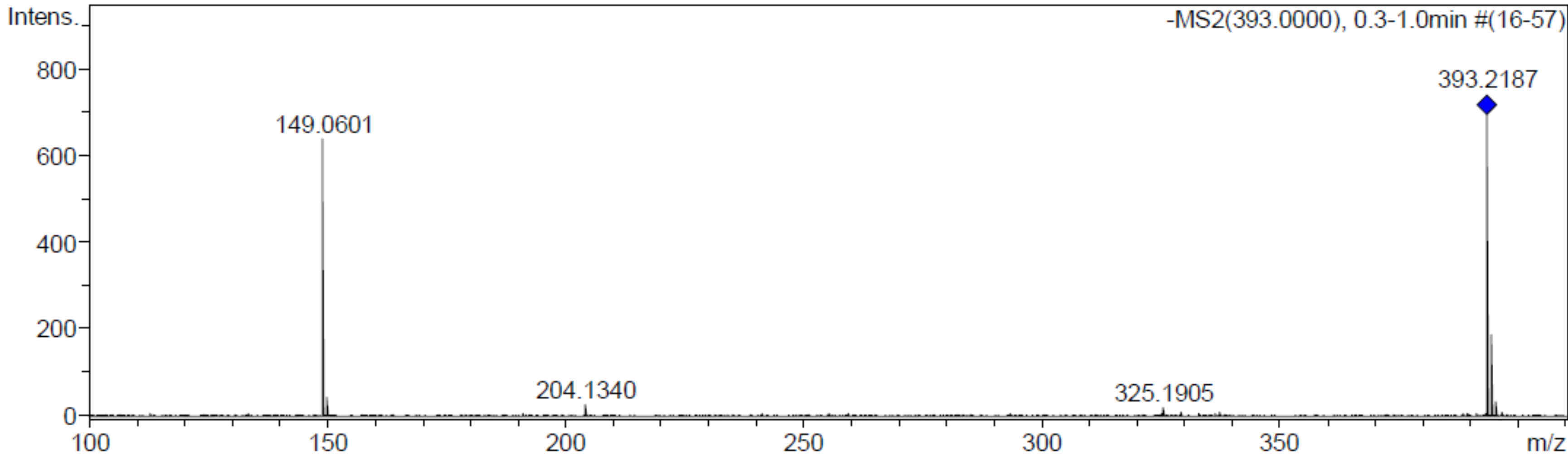

Supplement: Figure S5 — MS/MS of the deprotonated compound 5 measured by ESI-QTOF. (TIF) [file pone.0063097.s005.tif]

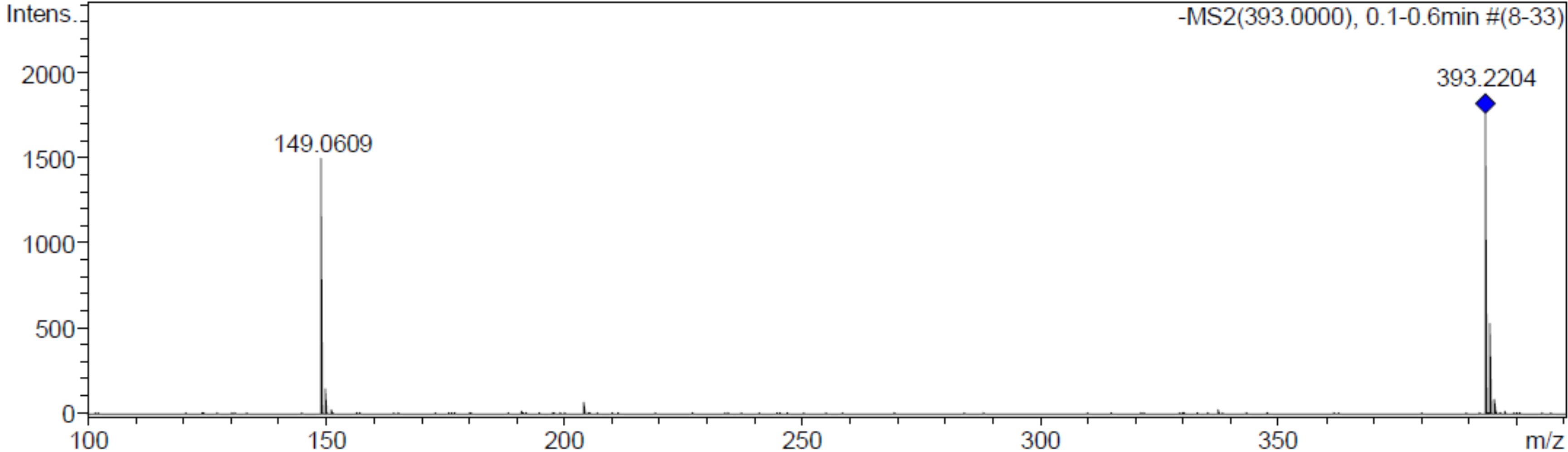

Supplement: Figure S6 — MS/MS of the deprotonated compound 6 measured by ESI-QTOF. (TIF) [file pone.0063097.s006.tif]
