# Supplementary material for: Fragmentation of Deprotonated Diacylhydrazine Derivatives in Electrospray Ionization Tandem Mass Spectrometry: Generation of Acid Anions via Intramolecular Rearrangement
Source: PLoS One. 2013 May 21;8(5):e63097. doi: 10.1371/journal.pone.0063097 (PMC3660572; doi:10.1371/journal.pone.0063097)
Supplement: Text S1 — Geometries for R01, R02-TS1, R03, R03-TSp, PI1, PN1, R01-TS2, R11, PN2, R11-TS, R12, R12-TS, R13, R13-TS, R14, R14-TSp, PI2, PN3. (DOC) [file pone.0063097.s007.doc]

**Table S1 Geometries for R01.**

| Center Number | Atomic Number | Atomic Type | X* | Y* | Z* |
| --- | --- | --- | --- | --- | --- |
| 1 | 6 | 0 | -4.403683 | -0.108247 | -0.224649 |
| 2 | 6 | 0 | -3.480084 | 0.034245 | -1.257109 |
| 3 | 6 | 0 | -2.126930 | -0.166732 | -0.985241 |
| 4 | 6 | 0 | -1.689980 | -0.504609 | 0.302890 |
| 5 | 6 | 0 | -2.644490 | -0.634809 | 1.316815 |
| 6 | 6 | 0 | -4.003652 | -0.441146 | 1.064823 |
| 7 | 1 | 0 | -3.814556 | 0.298892 | -2.254192 |
| 8 | 1 | 0 | -1.385971 | -0.061144 | -1.768644 |
| 9 | 1 | 0 | -2.290789 | -0.890864 | 2.309790 |
| 10 | 1 | 0 | -4.739274 | -0.545525 | 1.854848 |
| 11 | 6 | 0 | -0.226655 | -0.731432 | 0.651456 |
| 12 | 7 | 0 | 0.566603 | -0.638350 | -0.414691 |
| 13 | 7 | 0 | 1.943474 | -0.879197 | -0.122330 |
| 14 | 6 | 0 | 2.806552 | 0.161262 | -0.158420 |
| 15 | 6 | 0 | 2.240520 | 1.556419 | -0.036522 |
| 16 | 6 | 0 | 1.434594 | 1.947068 | 1.038824 |
| 17 | 6 | 0 | 2.656923 | 2.520456 | -0.960900 |
| 18 | 6 | 0 | 1.046706 | 3.280030 | 1.176213 |
| 19 | 1 | 0 | 1.112083 | 1.201692 | 1.759052 |
| 20 | 6 | 0 | 2.249846 | 3.848599 | -0.836263 |
| 21 | 1 | 0 | 3.310057 | 2.218527 | -1.773183 |
| 22 | 6 | 0 | 1.444381 | 4.234407 | 0.237192 |
| 23 | 1 | 0 | 0.423154 | 3.571670 | 2.016968 |
| 24 | 1 | 0 | 2.567600 | 4.582989 | -1.571824 |
| 25 | 1 | 0 | 1.130703 | 5.269614 | 0.341891 |
| 26 | 8 | 0 | 0.063841 | -0.962809 | 1.849246 |
| 27 | 8 | 0 | 4.039487 | 0.046588 | -0.272521 |
| 28 | 6 | 0 | 2.450688 | -2.290497 | -0.222640 |
| 29 | 6 | 0 | 3.153865 | -2.497578 | -1.581309 |
| 30 | 1 | 0 | 4.017406 | -1.837395 | -1.678110 |
| 31 | 1 | 0 | 3.488841 | -3.538688 | -1.672661 |
| 32 | 1 | 0 | 2.453953 | -2.291484 | -2.399001 |
| 33 | 6 | 0 | 3.410003 | -2.573168 | 0.950799 |
| 34 | 1 | 0 | 3.751545 | -3.615008 | 0.899361 |
| 35 | 1 | 0 | 4.279323 | -1.915607 | 0.920817 |
| 36 | 1 | 0 | 2.882350 | -2.426340 | 1.898294 |
| 37 | 6 | 0 | 1.276789 | -3.278211 | -0.123732 |
| 38 | 1 | 0 | 0.733866 | -3.151172 | 0.815224 |
| 39 | 1 | 0 | 0.576301 | -3.159468 | -0.953032 |
| 40 | 1 | 0 | 1.686715 | -4.294738 | -0.156831 |
| 41 | 17 | 0 | -6.126638 | 0.141194 | -0.562164 |

^*^: Coordinates (Angstroms)

**Table S2 Geometries for R02-TS1.**

| Center Number | Atomic Number | Atomic Type | X* | Y* | Z* |
| --- | --- | --- | --- | --- | --- |
| 1 | 6 | 0 | -4.840312 | -0.450475 | -0.078521 |
| 2 | 6 | 0 | -4.316366 | 0.789907 | -0.453175 |
| 3 | 6 | 0 | -2.944077 | 0.989474 | -0.429881 |
| 4 | 6 | 0 | -2.054035 | -0.036669 | -0.025310 |
| 5 | 6 | 0 | -2.620664 | -1.279969 | 0.341661 |
| 6 | 6 | 0 | -3.998196 | -1.484517 | 0.317639 |
| 7 | 1 | 0 | -4.981937 | 1.588960 | -0.762088 |
| 8 | 1 | 0 | -2.530350 | 1.948160 | -0.721338 |
| 9 | 1 | 0 | -1.962666 | -2.082173 | 0.653794 |
| 10 | 1 | 0 | -4.413951 | -2.444013 | 0.606497 |
| 11 | 6 | 0 | -0.634796 | 0.174697 | 0.008038 |
| 12 | 7 | 0 | -0.038361 | 1.304054 | -0.252260 |
| 13 | 7 | 0 | 1.277351 | 1.216262 | -0.206208 |
| 14 | 6 | 0 | 1.398280 | -0.592206 | 0.945264 |
| 15 | 6 | 0 | 2.559245 | -1.264348 | 0.256073 |
| 16 | 6 | 0 | 2.520507 | -1.667815 | -1.084009 |
| 17 | 6 | 0 | 3.713820 | -1.515267 | 1.003761 |
| 18 | 6 | 0 | 3.619522 | -2.297883 | -1.664462 |
| 19 | 1 | 0 | 1.626101 | -1.474018 | -1.662985 |
| 20 | 6 | 0 | 4.817144 | -2.142721 | 0.423771 |
| 21 | 1 | 0 | 3.724249 | -1.207211 | 2.043775 |
| 22 | 6 | 0 | 4.775216 | -2.536454 | -0.914407 |
| 23 | 1 | 0 | 3.576418 | -2.602752 | -2.706742 |
| 24 | 1 | 0 | 5.708259 | -2.326740 | 1.018265 |
| 25 | 1 | 0 | 5.632019 | -3.026048 | -1.369520 |
| 26 | 8 | 0 | 0.174195 | -0.915594 | 0.304393 |
| 27 | 8 | 0 | 1.420610 | -0.325543 | 2.136001 |
| 28 | 6 | 0 | 1.962736 | 2.514003 | -0.123035 |
| 29 | 6 | 0 | 3.469771 | 2.217344 | -0.125653 |
| 30 | 1 | 0 | 3.752016 | 1.667570 | -1.029887 |
| 31 | 1 | 0 | 4.046709 | 3.149803 | -0.088917 |
| 32 | 1 | 0 | 3.745366 | 1.605874 | 0.738828 |
| 33 | 6 | 0 | 1.584309 | 3.253674 | 1.180731 |
| 34 | 1 | 0 | 0.511695 | 3.471802 | 1.191375 |
| 35 | 1 | 0 | 1.809518 | 2.624991 | 2.047758 |
| 36 | 1 | 0 | 2.134603 | 4.200187 | 1.274953 |
| 37 | 6 | 0 | 1.610491 | 3.395890 | -1.340386 |
| 38 | 1 | 0 | 1.906070 | 2.895966 | -2.269959 |
| 39 | 1 | 0 | 0.532487 | 3.576305 | -1.379232 |
| 40 | 1 | 0 | 2.127808 | 4.363490 | -1.285800 |
| 41 | 17 | 0 | -6.594786 | -0.708427 | -0.120073 |

^*^: Coordinates (Angstroms)

**Table S3 Geometries for R03.**

| Center Number | Atomic Number | Atomic Type | X* | Y* | Z* |
| --- | --- | --- | --- | --- | --- |
| 1 | 6 | 0 | -4.479334 | -0.926711 | -0.059159 |
| 2 | 6 | 0 | -4.193695 | 0.440042 | 0.010282 |
| 3 | 6 | 0 | -2.876766 | 0.871558 | -0.001590 |
| 4 | 6 | 0 | -1.788499 | -0.042403 | -0.081983 |
| 5 | 6 | 0 | -2.123865 | -1.420968 | -0.148629 |
| 6 | 6 | 0 | -3.446477 | -1.855421 | -0.138133 |
| 7 | 1 | 0 | -5.003096 | 1.160297 | 0.073580 |
| 8 | 1 | 0 | -2.655223 | 1.930871 | 0.052638 |
| 9 | 1 | 0 | -1.329494 | -2.155687 | -0.208623 |
| 10 | 1 | 0 | -3.671495 | -2.915867 | -0.189451 |
| 11 | 6 | 0 | -0.435613 | 0.410493 | -0.099740 |
| 12 | 7 | 0 | -0.076611 | 1.674856 | -0.071554 |
| 13 | 7 | 0 | 1.174555 | 2.000588 | -0.132894 |
| 14 | 6 | 0 | 1.415911 | -0.857366 | 0.699353 |
| 15 | 6 | 0 | 2.609449 | -1.629968 | 0.215736 |
| 16 | 6 | 0 | 2.885519 | -1.804074 | -1.145460 |
| 17 | 6 | 0 | 3.469132 | -2.189240 | 1.167476 |
| 18 | 6 | 0 | 4.005376 | -2.532344 | -1.545881 |
| 19 | 1 | 0 | 2.223701 | -1.354710 | -1.875463 |
| 20 | 6 | 0 | 4.585829 | -2.920176 | 0.767215 |
| 21 | 1 | 0 | 3.242859 | -2.034604 | 2.216774 |
| 22 | 6 | 0 | 4.856814 | -3.094000 | -0.592132 |
| 23 | 1 | 0 | 4.216327 | -2.657617 | -2.604019 |
| 24 | 1 | 0 | 5.246143 | -3.351994 | 1.513895 |
| 25 | 1 | 0 | 5.728829 | -3.660890 | -0.906301 |
| 26 | 8 | 0 | 0.562201 | -0.592935 | -0.302903 |
| 27 | 8 | 0 | 1.249474 | -0.569530 | 1.863068 |
| 28 | 6 | 0 | 1.363143 | 3.468552 | -0.091452 |
| 29 | 6 | 0 | 2.879741 | 3.693595 | -0.178008 |
| 30 | 1 | 0 | 3.274434 | 3.268704 | -1.107951 |
| 31 | 1 | 0 | 3.126872 | 4.762870 | -0.148464 |
| 32 | 1 | 0 | 3.384487 | 3.193378 | 0.655785 |
| 33 | 6 | 0 | 0.826873 | 4.057814 | 1.229649 |
| 34 | 1 | 0 | -0.247341 | 3.868380 | 1.315983 |
| 35 | 1 | 0 | 1.322135 | 3.582368 | 2.083748 |
| 36 | 1 | 0 | 0.999351 | 5.141839 | 1.284603 |
| 37 | 6 | 0 | 0.668706 | 4.154363 | -1.286418 |
| 38 | 1 | 0 | 1.055268 | 3.752295 | -2.230498 |
| 39 | 1 | 0 | -0.408393 | 3.963902 | -1.254430 |
| 40 | 1 | 0 | 0.834816 | 5.240728 | -1.278473 |
| 41 | 17 | 0 | -6.167803 | -1.476772 | -0.045310 |

^*^: Coordinates (Angstroms)

**Table S4 Geometries for R03-TSp.**

| Center Number | Atomic Number | Atomic Type | X* | Y* | Z* |
| --- | --- | --- | --- | --- | --- |
| 1 | 6 | 0 | 4.440979 | -0.331584 | 0.198602 |
| 2 | 6 | 0 | 3.854724 | 0.849919 | 0.647107 |
| 3 | 6 | 0 | 2.501369 | 1.068017 | 0.419292 |
| 4 | 6 | 0 | 1.704288 | 0.111722 | -0.256582 |
| 5 | 6 | 0 | 2.329867 | -1.072839 | -0.698812 |
| 6 | 6 | 0 | 3.686136 | -1.289037 | -0.473146 |
| 7 | 1 | 0 | 4.449086 | 1.592560 | 1.167799 |
| 8 | 1 | 0 | 2.042764 | 1.986466 | 0.768446 |
| 9 | 1 | 0 | 1.726782 | -1.798548 | -1.226917 |
| 10 | 1 | 0 | 4.154603 | -2.202383 | -0.822838 |
| 11 | 6 | 0 | 0.315845 | 0.386585 | -0.436363 |
| 12 | 7 | 0 | -0.494049 | 1.276142 | -0.398780 |
| 13 | 7 | 0 | -1.639379 | 1.779150 | -0.630721 |
| 14 | 6 | 0 | -1.512761 | -1.758461 | -1.593787 |
| 15 | 6 | 0 | -2.265274 | -1.920254 | -0.272617 |
| 16 | 6 | 0 | -1.601206 | -1.951414 | 0.958012 |
| 17 | 6 | 0 | -3.655317 | -2.086711 | -0.287845 |
| 18 | 6 | 0 | -2.307785 | -2.143259 | 2.147331 |
| 19 | 1 | 0 | -0.524422 | -1.827770 | 0.972231 |
| 20 | 6 | 0 | -4.368232 | -2.262323 | 0.897276 |
| 21 | 1 | 0 | -4.154127 | -2.078358 | -1.250915 |
| 22 | 6 | 0 | -3.695464 | -2.294103 | 2.122292 |
| 23 | 1 | 0 | -1.774556 | -2.172627 | 3.094259 |
| 24 | 1 | 0 | -5.448973 | -2.377809 | 0.868109 |
| 25 | 1 | 0 | -4.247749 | -2.436416 | 3.047681 |
| 26 | 8 | 0 | -0.314324 | -1.307538 | -1.533756 |
| 27 | 8 | 0 | -2.095914 | -2.118309 | -2.625860 |
| 28 | 6 | 0 | -1.833603 | 3.113846 | -0.010386 |
| 29 | 6 | 0 | -3.262843 | 3.522257 | -0.390513 |
| 30 | 1 | 0 | -3.981146 | 2.790931 | -0.006234 |
| 31 | 1 | 0 | -3.510358 | 4.509143 | 0.018630 |
| 32 | 1 | 0 | -3.371125 | 3.554957 | -1.479679 |
| 33 | 6 | 0 | -0.829200 | 4.137833 | -0.576117 |
| 34 | 1 | 0 | 0.198725 | 3.844952 | -0.335987 |
| 35 | 1 | 0 | -0.917850 | 4.193037 | -1.666378 |
| 36 | 1 | 0 | -1.004866 | 5.138932 | -0.161007 |
| 37 | 6 | 0 | -1.702734 | 3.034519 | 1.523368 |
| 38 | 1 | 0 | -2.411729 | 2.304060 | 1.926620 |
| 39 | 1 | 0 | -0.693965 | 2.718409 | 1.810596 |
| 40 | 1 | 0 | -1.900856 | 4.008545 | 1.989400 |
| 41 | 17 | 0 | 6.164290 | -0.612743 | 0.486290 |

^*^: Coordinates (Angstroms)

**Table S5 Geometries for PI1.**

| Center Number | Atomic Number | Atomic Type | X* | Y* | Z* |
| --- | --- | --- | --- | --- | --- |
| 1 | 6 | 0 | 1.831034 | 0.000000 | 0.000000 |
| 2 | 6 | 0 | 0.280815 | 0.000000 | 0.000000 |
| 3 | 6 | 0 | -0.436814 | -1.201976 | -0.000005 |
| 4 | 6 | 0 | -0.436814 | 1.201977 | 0.000005 |
| 5 | 6 | 0 | -1.833542 | -1.206680 | -0.000005 |
| 6 | 1 | 0 | 0.136620 | -2.123529 | -0.000008 |
| 7 | 6 | 0 | -1.833542 | 1.206680 | 0.000006 |
| 8 | 1 | 0 | 0.136620 | 2.123530 | 0.000008 |
| 9 | 6 | 0 | -2.539600 | 0.000000 | 0.000000 |
| 10 | 1 | 0 | -2.374793 | -2.150812 | -0.000010 |
| 11 | 1 | 0 | -2.374794 | 2.150812 | 0.000010 |
| 12 | 1 | 0 | -3.627238 | 0.000000 | 0.000000 |
| 13 | 8 | 0 | 2.369647 | -1.133055 | 0.000013 |
| 14 | 8 | 0 | 2.369648 | 1.133055 | -0.000012 |

^*^: Coordinates (Angstroms)

**Table S6 Geometries for PN1.**

| Center Number | Atomic Number | Atomic Type | X* | Y* | Z* |
| --- | --- | --- | --- | --- | --- |
| 1 | 6 | 0 | -3.392766 | -0.106292 | 0.041951 |
| 2 | 6 | 0 | -2.468282 | -1.078362 | -0.341864 |
| 3 | 6 | 0 | -1.138409 | -0.716127 | -0.521290 |
| 4 | 6 | 0 | -0.722681 | 0.615101 | -0.329536 |
| 5 | 6 | 0 | -1.677188 | 1.573421 | 0.044723 |
| 6 | 6 | 0 | -3.008947 | 1.218099 | 0.239644 |
| 7 | 1 | 0 | -2.788560 | -2.102605 | -0.492910 |
| 8 | 1 | 0 | -0.413382 | -1.466636 | -0.818088 |
| 9 | 1 | 0 | -1.363862 | 2.602465 | 0.181538 |
| 10 | 1 | 0 | -3.742807 | 1.958917 | 0.533713 |
| 11 | 6 | 0 | 0.654290 | 1.018999 | -0.532736 |
| 12 | 7 | 0 | 1.698015 | 0.437293 | -0.677217 |
| 13 | 7 | 0 | 2.805767 | -0.021459 | -0.973135 |
| 14 | 6 | 0 | 3.759198 | -0.225902 | 0.173794 |
| 15 | 6 | 0 | 4.977152 | -0.917570 | -0.444100 |
| 16 | 1 | 0 | 5.407752 | -0.300234 | -1.238239 |
| 17 | 1 | 0 | 5.744854 | -1.091988 | 0.316724 |
| 18 | 1 | 0 | 4.694655 | -1.881096 | -0.879695 |
| 19 | 6 | 0 | 3.119731 | -1.114698 | 1.251793 |
| 20 | 1 | 0 | 2.233823 | -0.639329 | 1.685833 |
| 21 | 1 | 0 | 2.823913 | -2.081347 | 0.831302 |
| 22 | 1 | 0 | 3.831947 | -1.298833 | 2.063312 |
| 23 | 6 | 0 | 4.145615 | 1.144864 | 0.750781 |
| 24 | 1 | 0 | 4.604291 | 1.772323 | -0.019681 |
| 25 | 1 | 0 | 3.269623 | 1.670638 | 1.144954 |
| 26 | 1 | 0 | 4.863165 | 1.024160 | 1.569605 |
| 27 | 17 | 0 | -5.069304 | -0.561557 | 0.275007 |

^*^: Coordinates (Angstroms)

**Table S7 Geometries for R01-TS2.**

| Center Number | Atomic Number | Atomic Type | X* | Y* | Z* |
| --- | --- | --- | --- | --- | --- |
| 1 | 6 | 0 | 4.518944 | 0.205951 | 0.192274 |
| 2 | 6 | 0 | 3.443745 | 0.912618 | 0.725816 |
| 3 | 6 | 0 | 2.147435 | 0.467354 | 0.468182 |
| 4 | 6 | 0 | 1.912511 | -0.674393 | -0.312801 |
| 5 | 6 | 0 | 3.018278 | -1.360515 | -0.830647 |
| 6 | 6 | 0 | 4.323009 | -0.929256 | -0.586801 |
| 7 | 1 | 0 | 3.618509 | 1.794525 | 1.332447 |
| 8 | 1 | 0 | 1.293984 | 0.998569 | 0.872721 |
| 9 | 1 | 0 | 2.824475 | -2.243589 | -1.430018 |
| 10 | 1 | 0 | 5.173815 | -1.465430 | -0.993224 |
| 11 | 6 | 0 | 0.521783 | -1.201475 | -0.621658 |
| 12 | 7 | 0 | -0.437813 | -0.405344 | -0.137818 |
| 13 | 7 | 0 | -1.726637 | -0.813034 | -0.412956 |
| 14 | 6 | 0 | -2.657978 | 0.121656 | -0.375395 |
| 15 | 6 | 0 | -2.420791 | 1.599929 | -0.241346 |
| 16 | 6 | 0 | -1.304286 | 2.271099 | -0.765941 |
| 17 | 6 | 0 | -3.417875 | 2.366160 | 0.384821 |
| 18 | 6 | 0 | -1.190900 | 3.657362 | -0.657590 |
| 19 | 1 | 0 | -0.528038 | 1.698046 | -1.252884 |
| 20 | 6 | 0 | -3.293238 | 3.748462 | 0.513991 |
| 21 | 1 | 0 | -4.297348 | 1.854310 | 0.758715 |
| 22 | 6 | 0 | -2.176653 | 4.403935 | -0.008788 |
| 23 | 1 | 0 | -0.321510 | 4.155034 | -1.078899 |
| 24 | 1 | 0 | -4.072934 | 4.315202 | 1.016625 |
| 25 | 1 | 0 | -2.078044 | 5.482256 | 0.083539 |
| 26 | 8 | 0 | 0.436123 | -2.278415 | -1.262572 |
| 27 | 8 | 0 | -3.910754 | -0.231330 | -0.442497 |
| 28 | 6 | 0 | -2.534057 | -2.726222 | 0.626687 |
| 29 | 6 | 0 | -3.839412 | -2.343299 | 1.039417 |
| 30 | 1 | 0 | -3.998262 | -1.270947 | 0.329158 |
| 31 | 1 | 0 | -4.655713 | -2.942606 | 0.626660 |
| 32 | 1 | 0 | -3.965684 | -2.082590 | 2.091623 |
| 33 | 6 | 0 | -2.387230 | -3.582577 | -0.597624 |
| 34 | 1 | 0 | -2.451204 | -4.639447 | -0.285246 |
| 35 | 1 | 0 | -3.199701 | -3.391961 | -1.304591 |
| 36 | 1 | 0 | -1.426034 | -3.405099 | -1.085267 |
| 37 | 6 | 0 | -1.410064 | -2.751900 | 1.615856 |
| 38 | 1 | 0 | -0.436990 | -2.633647 | 1.140159 |
| 39 | 1 | 0 | -1.533116 | -1.988021 | 2.388212 |
| 40 | 1 | 0 | -1.427946 | -3.737824 | 2.108088 |
| 41 | 17 | 0 | 6.171213 | 0.764735 | 0.515731 |

^*^: Coordinates (Angstroms)

**Table S8 Geometries for R11.**

| Center Number | Atomic Number | Atomic Type | X* | Y* | Z* |
| --- | --- | --- | --- | --- | --- |
| 1 | 6 | 0 | 6.499813 | -1.095102 | -0.211719 |
| 2 | 6 | 0 | 5.655674 | -0.899277 | -1.309569 |
| 3 | 6 | 0 | 4.316425 | -0.558786 | -1.117256 |
| 4 | 6 | 0 | 3.797867 | -0.405292 | 0.177011 |
| 5 | 6 | 0 | 4.649574 | -0.607138 | 1.269652 |
| 6 | 6 | 0 | 5.989520 | -0.948924 | 1.080460 |
| 7 | 1 | 0 | 6.044398 | -1.016566 | -2.320134 |
| 8 | 1 | 0 | 3.644638 | -0.408548 | -1.955471 |
| 9 | 1 | 0 | 4.221260 | -0.488407 | 2.260291 |
| 10 | 1 | 0 | 6.638244 | -1.102579 | 1.941751 |
| 11 | 6 | 0 | 2.347874 | -0.030056 | 0.449914 |
| 12 | 7 | 0 | 1.673989 | 0.229326 | -0.673781 |
| 13 | 7 | 0 | 0.302459 | 0.525396 | -0.403750 |
| 14 | 6 | 0 | -0.537541 | -0.529045 | -0.561042 |
| 15 | 6 | 0 | -2.032980 | -0.426909 | -0.308970 |
| 16 | 6 | 0 | -2.552319 | -0.652639 | 0.973411 |
| 17 | 6 | 0 | -2.930283 | -0.366524 | -1.385136 |
| 18 | 6 | 0 | -3.926288 | -0.778174 | 1.187187 |
| 19 | 1 | 0 | -1.868945 | -0.740167 | 1.812796 |
| 20 | 6 | 0 | -4.306599 | -0.480690 | -1.190948 |
| 21 | 1 | 0 | -2.543935 | -0.232297 | -2.391714 |
| 22 | 6 | 0 | -4.790621 | -0.682876 | 0.099998 |
| 23 | 1 | 0 | -4.319258 | -0.951063 | 2.184057 |
| 24 | 1 | 0 | -4.993944 | -0.421282 | -2.028956 |
| 25 | 8 | 0 | 1.970801 | 0.031454 | 1.649271 |
| 26 | 8 | 0 | -0.178208 | -1.663014 | -0.899369 |
| 27 | 6 | 0 | -0.056563 | 1.946433 | -0.061175 |
| 28 | 6 | 0 | -1.062935 | 2.526252 | -1.078524 |
| 29 | 1 | 0 | -2.038180 | 2.038181 | -1.029914 |
| 30 | 1 | 0 | -1.218598 | 3.594101 | -0.877514 |
| 31 | 1 | 0 | -0.674112 | 2.424111 | -2.098555 |
| 32 | 6 | 0 | -0.595260 | 2.050153 | 1.381794 |
| 33 | 1 | 0 | -0.626408 | 3.102264 | 1.696724 |
| 34 | 1 | 0 | -1.608323 | 1.649975 | 1.473580 |
| 35 | 1 | 0 | 0.078467 | 1.491676 | 2.037296 |
| 36 | 6 | 0 | 1.222064 | 2.805693 | -0.139399 |
| 37 | 1 | 0 | 1.935634 | 2.511388 | 0.632431 |
| 38 | 1 | 0 | 1.706797 | 2.718062 | -1.113607 |
| 39 | 1 | 0 | 0.938526 | 3.853040 | 0.029489 |
| 40 | 17 | 0 | -6.538890 | -0.828995 | 0.360006 |
| 41 | 1 | 0 | 7.544467 | -1.362132 | -0.363305 |

^*^: Coordinates (Angstroms)

**Table S9 Geometries for PN2.**

| Center Number | Atomic Number | Atomic Type | X* | Y* | Z* |
| --- | --- | --- | --- | --- | --- |
| 1 | 6 | 0 | 4.192392 | -0.608852 | -0.046265 |
| 2 | 6 | 0 | 2.987845 | -1.237224 | -0.351582 |
| 3 | 6 | 0 | 1.808639 | -0.492953 | -0.309198 |
| 4 | 6 | 0 | 1.817493 | 0.867193 | 0.030487 |
| 5 | 6 | 0 | 3.048821 | 1.465915 | 0.324630 |
| 6 | 6 | 0 | 4.239423 | 0.739232 | 0.292958 |
| 7 | 1 | 0 | 2.974030 | -2.288110 | -0.619702 |
| 8 | 1 | 0 | 0.861307 | -0.959947 | -0.549374 |
| 9 | 1 | 0 | 3.043759 | 2.521198 | 0.575878 |
| 10 | 1 | 0 | 5.188415 | 1.210349 | 0.525291 |
| 11 | 6 | 0 | 0.567344 | 1.731836 | 0.074288 |
| 12 | 7 | 0 | -0.563710 | 1.004391 | -0.073406 |
| 13 | 7 | 0 | -1.674805 | 1.784418 | -0.130891 |
| 14 | 6 | 0 | -2.856083 | 1.275376 | -0.131264 |
| 15 | 6 | 0 | -3.352639 | -0.099750 | -0.017398 |
| 16 | 6 | 0 | -2.491465 | -1.191308 | 0.218611 |
| 17 | 6 | 0 | -4.735482 | -0.360225 | -0.131150 |
| 18 | 6 | 0 | -3.000080 | -2.483543 | 0.330374 |
| 19 | 1 | 0 | -1.433127 | -0.988849 | 0.309230 |
| 20 | 6 | 0 | -5.233731 | -1.655770 | -0.020469 |
| 21 | 1 | 0 | -5.410439 | 0.468007 | -0.307433 |
| 22 | 6 | 0 | -4.370079 | -2.729482 | 0.210608 |
| 23 | 1 | 0 | -2.315950 | -3.307737 | 0.515298 |
| 24 | 1 | 0 | -6.303108 | -1.827531 | -0.114197 |
| 25 | 1 | 0 | -4.758857 | -3.740425 | 0.297502 |
| 26 | 8 | 0 | 0.712186 | 2.955112 | 0.249429 |
| 27 | 8 | 0 | -3.883177 | 2.207758 | -0.261170 |
| 28 | 1 | 0 | -3.405493 | 3.049590 | -0.319648 |
| 29 | 17 | 0 | 5.697802 | -1.546108 | -0.096272 |

^*^: Coordinates (Angstroms)

**Table S10 Geometries for R11-TS.**

| Center Number | Atomic Number | Atomic Type | X* | Y* | Z* |
| --- | --- | --- | --- | --- | --- |
| 1 | 6 | 0 | -4.755477 | -2.221584 | 0.000000 |
| 2 | 6 | 0 | -3.657871 | -1.366500 | -0.000034 |
| 3 | 6 | 0 | -3.828991 | 0.028431 | -0.000017 |
| 4 | 6 | 0 | -5.137797 | 0.540262 | 0.000035 |
| 5 | 6 | 0 | -6.237934 | -0.319330 | 0.000071 |
| 6 | 6 | 0 | -6.055758 | -1.703380 | 0.000053 |
| 7 | 1 | 0 | -4.599919 | -3.297415 | -0.000016 |
| 8 | 1 | 0 | -2.643586 | -1.753588 | -0.000070 |
| 9 | 1 | 0 | -5.276736 | 1.614428 | 0.000047 |
| 10 | 1 | 0 | -7.243156 | 0.095191 | 0.000113 |
| 11 | 1 | 0 | -6.913138 | -2.370922 | 0.000079 |
| 12 | 6 | 0 | -2.619372 | 0.890270 | -0.000048 |
| 13 | 7 | 0 | -1.444067 | 0.526234 | -0.000034 |
| 14 | 7 | 0 | -0.206667 | 0.020698 | -0.000025 |
| 15 | 6 | 0 | 0.737626 | 1.004748 | 0.000007 |
| 16 | 6 | 0 | 2.146772 | 0.452557 | 0.000008 |
| 17 | 6 | 0 | 3.218862 | 1.352759 | 0.000051 |
| 18 | 6 | 0 | 2.429774 | -0.921226 | -0.000036 |
| 19 | 6 | 0 | 4.541330 | 0.907990 | 0.000051 |
| 20 | 1 | 0 | 2.986896 | 2.412326 | 0.000085 |
| 21 | 6 | 0 | 3.744913 | -1.385238 | -0.000038 |
| 22 | 1 | 0 | 1.598899 | -1.616244 | -0.000068 |
| 23 | 6 | 0 | 4.786942 | -0.461023 | 0.000006 |
| 24 | 1 | 0 | 5.367475 | 1.610702 | 0.000085 |
| 25 | 1 | 0 | 3.960089 | -2.448257 | -0.000072 |
| 26 | 8 | 0 | -2.988175 | 2.278924 | -0.000092 |
| 27 | 8 | 0 | 0.577375 | 2.238512 | 0.000038 |
| 28 | 1 | 0 | -2.124300 | 2.721507 | -0.000081 |
| 29 | 17 | 0 | 6.461818 | -1.042833 | 0.000005 |

^*^: Coordinates (Angstroms)

**Table S11 Geometries for R12.**

| Center Number | Atomic Number | Atomic Type | X* | Y* | Z* |
| --- | --- | --- | --- | --- | --- |
| 1 | 6 | 0 | -4.806573 | -0.253738 | -0.000001 |
| 2 | 6 | 0 | -3.802152 | -1.217968 | 0.000007 |
| 3 | 6 | 0 | -2.469697 | -0.805096 | 0.000009 |
| 4 | 6 | 0 | -2.127634 | 0.554280 | 0.000003 |
| 5 | 6 | 0 | -3.165642 | 1.494476 | -0.000005 |
| 6 | 6 | 0 | -4.504853 | 1.104047 | -0.000007 |
| 7 | 1 | 0 | -4.058537 | -2.271837 | 0.000012 |
| 8 | 1 | 0 | -1.676549 | -1.542235 | 0.000015 |
| 9 | 1 | 0 | -2.887943 | 2.543009 | -0.000009 |
| 10 | 1 | 0 | -5.301538 | 1.840116 | -0.000014 |
| 11 | 6 | 0 | -0.696622 | 1.072058 | 0.000005 |
| 12 | 7 | 0 | 0.219688 | 0.082044 | 0.000002 |
| 13 | 7 | 0 | 1.520590 | 0.517285 | 0.000002 |
| 14 | 6 | 0 | 2.350889 | -0.476494 | 0.000000 |
| 15 | 6 | 0 | 3.799365 | -0.280902 | -0.000001 |
| 16 | 6 | 0 | 4.346561 | 1.017199 | -0.000001 |
| 17 | 6 | 0 | 4.677534 | -1.378881 | -0.000001 |
| 18 | 6 | 0 | 5.723270 | 1.203625 | -0.000002 |
| 19 | 1 | 0 | 3.662877 | 1.858708 | -0.000001 |
| 20 | 6 | 0 | 6.059537 | -1.184189 | -0.000002 |
| 21 | 1 | 0 | 4.263509 | -2.380620 | -0.000001 |
| 22 | 6 | 0 | 6.593158 | 0.104856 | -0.000003 |
| 23 | 1 | 0 | 6.125878 | 2.213185 | -0.000003 |
| 24 | 1 | 0 | 6.721629 | -2.046389 | -0.000002 |
| 25 | 1 | 0 | 7.669067 | 0.255353 | -0.000003 |
| 26 | 8 | 0 | -0.524475 | 2.302865 | 0.000008 |
| 27 | 8 | 0 | 1.907522 | -1.766405 | -0.000002 |
| 28 | 1 | 0 | 0.928477 | -1.609567 | 0.000000 |
| 29 | 17 | 0 | -6.503297 | -0.768609 | -0.000004 |

^*^: Coordinates (Angstroms)

**Table S12 Geometries for R12-TS.**

| Center Number | Atomic Number | Atomic Type | X* | Y* | Z* |
| --- | --- | --- | --- | --- | --- |
| 1 | 6 | 0 | 5.842352 | 0.986954 | 0.000133 |
| 2 | 6 | 0 | 4.452067 | 0.956825 | 0.000117 |
| 3 | 6 | 0 | 3.765090 | -0.270376 | -0.000002 |
| 4 | 6 | 0 | 4.509664 | -1.460133 | -0.000105 |
| 5 | 6 | 0 | 5.904803 | -1.425308 | -0.000089 |
| 6 | 6 | 0 | 6.579895 | -0.203898 | 0.000030 |
| 7 | 1 | 0 | 6.358422 | 1.943555 | 0.000226 |
| 8 | 1 | 0 | 3.868876 | 1.871033 | 0.000195 |
| 9 | 1 | 0 | 3.974346 | -2.403022 | -0.000198 |
| 10 | 1 | 0 | 6.465956 | -2.356329 | -0.000171 |
| 11 | 1 | 0 | 7.666108 | -0.176245 | 0.000042 |
| 12 | 6 | 0 | 2.296940 | -0.317065 | -0.000021 |
| 13 | 7 | 0 | 1.582464 | 0.787435 | 0.000061 |
| 14 | 7 | 0 | 0.268191 | 0.357616 | 0.000038 |
| 15 | 6 | 0 | -0.725929 | 1.257753 | -0.000023 |
| 16 | 6 | 0 | -2.112411 | 0.634916 | -0.000007 |
| 17 | 6 | 0 | -3.211466 | 1.502877 | -0.000096 |
| 18 | 6 | 0 | -2.361118 | -0.744759 | 0.000108 |
| 19 | 6 | 0 | -4.520696 | 1.022489 | -0.000088 |
| 20 | 1 | 0 | -3.005430 | 2.567699 | -0.000179 |
| 21 | 6 | 0 | -3.662742 | -1.245941 | 0.000115 |
| 22 | 1 | 0 | -1.530679 | -1.440296 | 0.000213 |
| 23 | 6 | 0 | -4.730394 | -0.352448 | 0.000015 |
| 24 | 1 | 0 | -5.364939 | 1.703306 | -0.000158 |
| 25 | 1 | 0 | -3.845569 | -2.314788 | 0.000202 |
| 26 | 8 | 0 | 1.666303 | -1.480034 | -0.000125 |
| 27 | 8 | 0 | -0.632215 | 2.495289 | -0.000094 |
| 28 | 1 | 0 | 0.607652 | -0.915107 | -0.000092 |
| 29 | 17 | 0 | -6.386963 | -0.980501 | 0.000027 |

^*^: Coordinates (Angstroms)

**Table S13 Geometries for R13.**

| Center Number | Atomic Number | Atomic Type | X^*^ | Y^*^ | Z^*^ |
| --- | --- | --- | --- | --- | --- |
| 1 | 6 | 0 | -5.765769 | 1.107764 | 0.126728 |
| 2 | 6 | 0 | -4.380273 | 0.961640 | 0.100386 |
| 3 | 6 | 0 | -3.798734 | -0.309008 | -0.031652 |
| 4 | 6 | 0 | -4.639393 | -1.424528 | -0.136443 |
| 5 | 6 | 0 | -6.027496 | -1.278935 | -0.110239 |
| 6 | 6 | 0 | -6.598552 | -0.011888 | 0.021635 |
| 7 | 1 | 0 | -6.200741 | 2.098865 | 0.229813 |
| 8 | 1 | 0 | -3.723632 | 1.820438 | 0.180719 |
| 9 | 1 | 0 | -4.173165 | -2.398675 | -0.237937 |
| 10 | 1 | 0 | -6.664673 | -2.156185 | -0.193032 |
| 11 | 1 | 0 | -7.679109 | 0.104390 | 0.042300 |
| 12 | 6 | 0 | -2.309780 | -0.514000 | -0.063373 |
| 13 | 7 | 0 | -1.596916 | 0.611269 | 0.039172 |
| 14 | 7 | 0 | -0.258872 | 0.298564 | 0.008797 |
| 15 | 6 | 0 | 0.737798 | 1.200430 | 0.059578 |
| 16 | 6 | 0 | 2.128380 | 0.601557 | 0.038753 |
| 17 | 6 | 0 | 3.188929 | 1.472969 | -0.244683 |
| 18 | 6 | 0 | 2.432402 | -0.739486 | 0.317675 |
| 19 | 6 | 0 | 4.508832 | 1.028285 | -0.273763 |
| 20 | 1 | 0 | 2.946184 | 2.512577 | -0.434596 |
| 21 | 6 | 0 | 3.748137 | -1.201917 | 0.296990 |
| 22 | 1 | 0 | 1.647425 | -1.442788 | 0.574961 |
| 23 | 6 | 0 | 4.774030 | -0.310726 | -0.002994 |
| 24 | 1 | 0 | 5.321090 | 1.710270 | -0.499623 |
| 25 | 1 | 0 | 3.972579 | -2.239039 | 0.518796 |
| 26 | 8 | 0 | -1.841282 | -1.684957 | -0.178282 |
| 27 | 8 | 0 | 0.600052 | 2.427384 | 0.112731 |
| 28 | 17 | 0 | 6.445239 | -0.888912 | -0.027880 |
| 29 | 1 | 0 | -0.085746 | -0.699529 | -0.110416 |

^*^: Coordinates (Angstroms)

**Table S14 Geometries for R13-TS.**

| Center Number | Atomic Number | Atomic Type | X* | Y* | Z* |
| --- | --- | --- | --- | --- | --- |
| 1 | 6 | 0 | 0.003356 | 0.033718 | 0.007806 |
| 2 | 6 | 0 | 0.004450 | 0.019820 | 1.410960 |
| 3 | 6 | 0 | 1.193744 | -0.000622 | 2.125320 |
| 4 | 6 | 0 | 2.445761 | -0.016157 | 1.460719 |
| 5 | 6 | 0 | 2.431782 | 0.002345 | 0.046353 |
| 6 | 6 | 0 | 1.230926 | 0.025049 | -0.659387 |
| 7 | 1 | 0 | -0.939237 | 0.027398 | 1.951969 |
| 8 | 1 | 0 | 1.187166 | -0.008156 | 3.209646 |
| 9 | 1 | 0 | 3.377419 | -0.008558 | -0.483125 |
| 10 | 1 | 0 | 1.256170 | 0.034909 | -1.746765 |
| 11 | 6 | 0 | 3.679334 | -0.043829 | 2.196613 |
| 12 | 7 | 0 | 3.791449 | -0.124764 | 3.491683 |
| 13 | 7 | 0 | 5.045503 | -0.073161 | 3.911304 |
| 14 | 6 | 0 | 5.962360 | -0.632420 | 1.993593 |
| 15 | 6 | 0 | 7.187067 | 0.233647 | 2.113535 |
| 16 | 6 | 0 | 7.138741 | 1.632150 | 2.083022 |
| 17 | 6 | 0 | 8.429740 | -0.393662 | 2.238263 |
| 18 | 6 | 0 | 8.303054 | 2.390422 | 2.183592 |
| 19 | 1 | 0 | 6.177057 | 2.120833 | 1.992285 |
| 20 | 6 | 0 | 9.606161 | 0.349108 | 2.346386 |
| 21 | 1 | 0 | 8.456323 | -1.477687 | 2.251909 |
| 22 | 6 | 0 | 9.526130 | 1.736795 | 2.316807 |
| 23 | 1 | 0 | 8.263999 | 3.473743 | 2.162824 |
| 24 | 1 | 0 | 10.568093 | -0.141040 | 2.447259 |
| 25 | 8 | 0 | 4.872611 | 0.075137 | 1.468815 |
| 26 | 8 | 0 | 6.035916 | -1.844707 | 1.902593 |
| 27 | 1 | 0 | 5.076106 | -0.375681 | 4.886068 |
| 28 | 17 | 0 | 11.010731 | 2.695136 | 2.446924 |
| 29 | 1 | 0 | -0.930643 | 0.053118 | -0.546179 |

^*^: Coordinates (Angstroms)

**Table S15 Geometries for R14.**

| Center Number | Atomic Number | Atomic Type | X* | Y* | Z* |
| --- | --- | --- | --- | --- | --- |
| 1 | 6 | 0 | -5.385895 | -1.746591 | -0.019208 |
| 2 | 6 | 0 | -5.536891 | -0.366356 | -0.228341 |
| 3 | 6 | 0 | -4.437820 | 0.475086 | -0.307761 |
| 4 | 6 | 0 | -3.112148 | -0.024763 | -0.178732 |
| 5 | 6 | 0 | -2.977060 | -1.421413 | 0.034994 |
| 6 | 6 | 0 | -4.090248 | -2.254613 | 0.110200 |
| 7 | 1 | 0 | -6.533984 | 0.057547 | -0.330531 |
| 8 | 1 | 0 | -4.568192 | 1.539104 | -0.469098 |
| 9 | 1 | 0 | -1.983735 | -1.842398 | 0.143004 |
| 10 | 1 | 0 | -3.940705 | -3.319647 | 0.275830 |
| 11 | 6 | 0 | -1.979817 | 0.843327 | -0.267295 |
| 12 | 7 | 0 | -2.039031 | 2.134224 | -0.493175 |
| 13 | 7 | 0 | -0.935890 | 2.827145 | -0.595249 |
| 14 | 6 | 0 | 0.135610 | 0.486392 | 0.767914 |
| 15 | 6 | 0 | 1.562149 | 0.177962 | 0.411671 |
| 16 | 6 | 0 | 1.966554 | -0.074736 | -0.903533 |
| 17 | 6 | 0 | 2.513929 | 0.146848 | 1.435288 |
| 18 | 6 | 0 | 3.299612 | -0.361058 | -1.194337 |
| 19 | 1 | 0 | 1.229867 | -0.029815 | -1.695898 |
| 20 | 6 | 0 | 3.849584 | -0.141107 | 1.162549 |
| 21 | 1 | 0 | 2.187787 | 0.357401 | 2.447839 |
| 22 | 6 | 0 | 4.224449 | -0.393411 | -0.154303 |
| 23 | 1 | 0 | 3.617968 | -0.551069 | -2.212848 |
| 24 | 1 | 0 | 4.588461 | -0.167715 | 1.955211 |
| 25 | 8 | 0 | -0.691815 | 0.210331 | -0.248772 |
| 26 | 8 | 0 | -0.188837 | 0.872194 | 1.868914 |
| 27 | 17 | 0 | 5.916059 | -0.758562 | -0.514113 |
| 28 | 1 | 0 | -6.251257 | -2.399893 | 0.042305 |
| 29 | 1 | 0 | -1.241605 | 3.788844 | -0.772684 |

^*^: Coordinates (Angstroms)

**Table S16 Geometries for R14-TSp.**

| Center Number | Atomic Number | Atomic Type | X* | Y* | Z* |
| --- | --- | --- | --- | --- | --- |
| 1 | 6 | 0 | -0.014584 | -0.036219 | 0.032758 |
| 2 | 6 | 0 | 0.023545 | 0.045746 | 1.428636 |
| 3 | 6 | 0 | 1.237520 | 0.075352 | 2.104268 |
| 4 | 6 | 0 | 2.465146 | 0.026915 | 1.398463 |
| 5 | 6 | 0 | 2.416348 | -0.058134 | -0.008266 |
| 6 | 6 | 0 | 1.191457 | -0.088987 | -0.670249 |
| 7 | 1 | 0 | -0.901529 | 0.086230 | 1.998087 |
| 8 | 1 | 0 | 1.259293 | 0.139508 | 3.186945 |
| 9 | 1 | 0 | 3.352154 | -0.109885 | -0.547255 |
| 10 | 1 | 0 | 1.182597 | -0.158430 | -1.754911 |
| 11 | 6 | 0 | 3.681019 | 0.078893 | 2.149229 |
| 12 | 7 | 0 | 4.081605 | 0.011702 | 3.285601 |
| 13 | 7 | 0 | 5.059717 | -0.076121 | 4.114647 |
| 14 | 6 | 0 | 6.314424 | 0.358806 | 0.809944 |
| 15 | 6 | 0 | 6.462032 | 1.783826 | 1.339472 |
| 16 | 6 | 0 | 5.413884 | 2.706679 | 1.284258 |
| 17 | 6 | 0 | 7.695141 | 2.203552 | 1.849535 |
| 18 | 6 | 0 | 5.584004 | 4.021556 | 1.723436 |
| 19 | 1 | 0 | 4.455625 | 2.390918 | 0.888745 |
| 20 | 6 | 0 | 7.882653 | 3.505100 | 2.311103 |
| 21 | 1 | 0 | 8.506921 | 1.485070 | 1.873015 |
| 22 | 6 | 0 | 6.818975 | 4.400654 | 2.237025 |
| 23 | 1 | 0 | 4.770756 | 4.736852 | 1.671223 |
| 24 | 1 | 0 | 8.835955 | 3.823140 | 2.718566 |
| 25 | 8 | 0 | 5.124256 | -0.107059 | 0.701696 |
| 26 | 8 | 0 | 7.351069 | -0.226901 | 0.472259 |
| 27 | 1 | 0 | -0.964359 | -0.059540 | -0.493226 |
| 28 | 17 | 0 | 7.047416 | 6.064660 | 2.806278 |
| 29 | 1 | 0 | 4.653161 | -0.008572 | 5.053804 |

^*^: Coordinates (Angstroms)

**Table S17 Geometries for PI2.**

| Center Number | Atomic Number | Atomic Type | X* | Y* | Z* |
| --- | --- | --- | --- | --- | --- |
| 1 | 6 | 0 | 0.000000 | 0.000000 | -2.707667 |
| 2 | 6 | 0 | 0.000000 | 0.000000 | -1.156980 |
| 3 | 6 | 0 | 0.000000 | 1.200896 | -0.439839 |
| 4 | 6 | 0 | 0.000000 | -1.200896 | -0.439839 |
| 5 | 6 | 0 | 0.000000 | 1.215089 | 0.956838 |
| 6 | 1 | 0 | 0.000000 | 2.123777 | -1.010883 |
| 7 | 6 | 0 | 0.000000 | -1.215089 | 0.956838 |
| 8 | 1 | 0 | 0.000000 | -2.123777 | -1.010883 |
| 9 | 6 | 0 | 0.000000 | 0.000000 | 1.635223 |
| 10 | 1 | 0 | 0.000000 | 2.147911 | 1.511040 |
| 11 | 1 | 0 | 0.000000 | -2.147911 | 1.511040 |
| 12 | 8 | 0 | 0.000000 | 1.133812 | -3.241585 |
| 13 | 8 | 0 | 0.000000 | -1.133812 | -3.241585 |
| 14 | 17 | 0 | 0.000000 | 0.000000 | 3.413977 |

^*^: Coordinates (Angstroms)

**Table S18 Geometries for PN3.**

| Center Number | Atomic Number | Atomic Type | X* | Y* | Z* |
| --- | --- | --- | --- | --- | --- |
| 1 | 6 | 0 | 0.001194 | -0.000009 | 0.000747 |
| 2 | 1 | 0 | -0.001450 | 0.000395 | 1.087007 |
| 3 | 1 | 0 | 0.972147 | -0.000207 | -0.486307 |
| 4 | 6 | 0 | -1.135972 | -0.000249 | -0.702952 |
| 5 | 6 | 0 | -1.146715 | -0.000807 | -2.210401 |
| 6 | 1 | 0 | -1.677198 | -0.880638 | -2.597991 |
| 7 | 1 | 0 | -1.677155 | 0.878764 | -2.598642 |
| 8 | 1 | 0 | -0.134971 | -0.000985 | -2.625106 |
| 9 | 6 | 0 | -2.489832 | 0.000021 | -0.039945 |
| 10 | 1 | 0 | -3.073669 | 0.879617 | -0.341916 |
| 11 | 1 | 0 | -3.073695 | -0.879785 | -0.341254 |
| 12 | 1 | 0 | -2.409526 | 0.000429 | 1.050540 |

^*^: Coordinates (Angstroms)
